# Genomic and functional genomics analyses of gluten proteins and prospect for simultaneous improvement of end-use and health-related traits in wheat

**DOI:** 10.1007/s00122-020-03557-5

**Published:** 2020-02-04

**Authors:** Daowen Wang, Feng Li, Shuanghe Cao, Kunpu Zhang

**Affiliations:** 1 College of Agronomy, State Key Laboratory of Wheat and Maize Crop Science, and Center for Crop Genome Engineering, Henan Agricultural University, 15 Longzi Lake College Park, Zhengzhou, 450046 China; 2 State Key Laboratory of Plant Cell and Chromosome Engineering, Institute of Genetics and Developmental Biology, Chinese Academy of Science, 1 West Beichen Road, Beijing, 100101 China; 3 Institute of Crop Science, National Wheat Improvement Center, Chinese Academy of Agricultural Sciences, 12 Zhongguancun South Street, Beijing, 100081 China

## Abstract

**Key message:**

Recent genomic and functional genomics analyses have substantially improved the understanding on gluten proteins, which are important determinants of wheat grain quality traits. The new insights obtained and the availability of precise, versatile and high-throughput genome editing technologies will accelerate simultaneous improvement of wheat end-use and health-related traits.

**Abstract:**

Being a major staple food crop in the world, wheat provides an indispensable source of dietary energy and nutrients to the human population. As worldwide population grows and living standards rise in both developed and developing countries, the demand for wheat with high quality attributes increases globally. However, efficient breeding of high-quality wheat depends on critically the knowledge on gluten proteins, which mainly include several families of prolamin proteins specifically accumulated in the endospermic tissues of grains. Although gluten proteins have been studied for many decades, efficient manipulation of these proteins for simultaneous enhancement of end-use and health-related traits has been difficult because of high complexities in their expression, function and genetic variation. However, recent genomic and functional genomics analyses have substantially improved the understanding on gluten proteins. Therefore, the main objective of this review is to summarize the genomic and functional genomics information obtained in the last 10 years on gluten protein chromosome loci and genes and the *cis*- and *trans*-factors regulating their expression in the grains, as well as the efforts in elucidating the involvement of gluten proteins in several wheat sensitivities affecting genetically susceptible human individuals. The new insights gathered, plus the availability of precise, versatile and high-throughput genome editing technologies, promise to speed up the concurrent improvement of wheat end-use and health-related traits and the development of high-quality cultivars for different consumption needs.

## Introduction

Wheat (*Triticum aestivum*) is the most widely cultivated staple food crop in the world, providing approximately 20% of the total dietary calories and proteins globally and a wealth of additional health promoting nutrients to the daily human diet (Brouns et al. 2019; Shiferaw et al. 2013; Shewry and Hey 2015). Owing to its importance, global wheat production has risen significantly from 440.2 million tons in 1980 to 771.7 million tons in 2017 (FAOSTAT, http://www.fao.org/faostat/en/). As world population is expected to reach 9.8 billion by 2050, food production has to increase by at least 50% compared to the current level (FAO 2017). Furthermore, consumer demand of healthier foods is increasing worldwide because of rising living standards. Consequently, substantial efforts have to be devoted to improve the yield and quality traits of major agricultural crops including wheat.

Traditionally, wheat quality refers mainly to the end-use properties of the flour (Delcour et al. 2012; Gras et al. 2001; Rasheed et al. 2014; Wrigley et al. 2009). This aspect has been extensively studied since the first description of wheat gluten in 1745 (Ma et al. 2019; Shewry 2019). Typical wheat gluten proteins include several families of structurally similar and yet distinctive prolamin proteins, i.e., high and low molecular weight glutenin subunits (HMW-GSs and LMW-GSs) and gliadins (Table 1), with similar proteins present in related Triticeae species such as rye and barley (Shewry et al. 2003). The gluten proteins in each of the families generally have two or more types (Table 1). In HMW-GSs, there exist x- and y-type subunits; in LMW-GSs, i-, m- and s-types are found; for gliadins, α-, γ-, δ- and ω-types are differentiated. A common structural feature shared by different glutenins and gliadins is the presence of a repetitive domain composed of repetitive motifs rich in glutamine (Q) and proline (P) residues (Table 1). Another important characteristic shared by the gluten proteins is that they are specifically expressed in the developing grains and accumulate to relatively high amounts in the endospermic tissues (Shewry et al. 2003); in wheat cultivars, gluten proteins generally account for ~ 80% of the total grain proteins (Altenbach 2017).

**Table 1:** Main characteristics of wheat gluten proteins

Gluten protein	Type	Molecular mass (kDa)	Partial amino acid composition (%)			% in total gluten^b^	Polymeric or monomeric
			Glutamine	Proline	Cysteine		
HMW-GSs	x-type	83–88	30–35	10–16	0.5–1.5	9.3	Polymeric
	y-type	67–74					
LMW-GSs	i-type	32–35	30–45	15–25	2–3	22.3	Polymeric
	m-type						
	s-type						
Gliadins	α-type	28–35	30–40	15–20	2–3	53.4	Monomeric^d^
	γ-type	31–35					
	δ-type	32–36^a^					
	ω-type	44–74	40–50	20–30	–^c^	13.2	

Based on the data presented in D’Ovidio and Masci (2004), She et al. (2011), Shewry (2019) and Wieser (2007)

^a^According to Anderson et al. (2012) and Wan et al. (2013)

^b^According to Schalk et al. (2017)

^c^Although most ω-gliadins do not contain cysteine, a few expressed ω-gliadins have recently been found to carry one cysteine in their deduced proteins (Vensel et al. 2014; Wang et al. 2017)

^d^Some of the α-, ω- and γ-gliadins carrying an odd number of cysteine residues may be incorporated into glutenin polymers through disulfide bonding (Ferrante et al. 2006; Vensel et al. 2014)

At the desiccation stage of wheat grain development, HMW-GSs and LMW-GSs form glutenin polymers through intra- and inter-molecular disulfide bonds (Table 1), with HMW-GSs as backbone and LMW-GSs as branches (Naeem and MacRitchie 2005; van Herpen et al. 2008a; Wrigley et al. 2009). During dough processing, glutenin polymers interact with monomeric gliadins and other proteins to form gluten polymers of various sizes, thus conferring viscoelasticity to the dough (MacRitchie 2014). Among the gluten polymers, only those with a molecular mass ≥ 250 kDa contribute significantly and positively to dough functionality and end-use properties (Bangur et al. 1997; Tronsmo et al. 2002). Depending on method of analysis, the large-sized gluten polymers can be prepared as glutenin macropolymers (GMPs) or unextractable polymeric protein (UPP) complexes (Don et al. 2003a, b; Gupta et al. 1993). Don et al. (2006) showed that GMPs and UPP complexes are highly and positively correlated in both quantity and effects on dough functionality. These data, together with the findings from many genetic studies, support a model that glutenins and gliadins are the main determinants of dough viscoelasticity and thus the end-use properties of wheat flour. Compared to gliadins, HMW-GSs and LMW-GSs contribute more significantly to both dough elasticity and extensibility. In addition to glutenins and gliadins, recent studies suggest that farinins (b-type avenin-like proteins) and purinins (LMW gliadins) may also be regarded as gluten proteins because of their participation in gluten polymers and influences on dough functionality and end-use quality (Kasarda et al. 2013; Shewry 2019) (see below).

Apart from functioning in end-use quality, gluten proteins are also known to be involved in a number of wheat food sensitivities, including celiac disease (CD), IgE-mediated wheat allergy and nonceliac wheat sensitivity (NCWS) (Cabanillas 2019; Scherf et al. 2016). Based on current understanding, these diseases may be defined as adverse reactions to gluten and related proteins by the immune system of genetically susceptible human individuals. CD is an autoimmune disease, with an incidence of 1–3% in the human population (Lionetti et al. 2015). The T cell epitopes triggering CD have been detected in a variety of gluten proteins from wheat and similar proteins in rye and barley, with the most immunogenic and toxic types located in wheat α- and ω-gliadins (Juhász et al. 2018; Scherf et al. 2016). CD causes damage to the small intestine and results in a plethora of symptoms including malabsorption of nutrients (Lionetti et al. 2015). Wheat-dependent exercise-induced anaphylaxis (WDEIA) and baker’s asthma are two commonly encountered IgE-mediated wheat allergies, with the incidences estimated to range from 0.33 to 1.17% (Cabanillas 2019). The ω-5 gliadins and HMW-GSs are major allergens associated with WDEIA (Altenbach et al. 2018; Matsuo et al. 2004, 2005). However, many other wheat grain proteins, e.g., α-amylase/trypsin inhibitors (ATIs) and nonspecific lipid transfer proteins (nsLTPs), may also be involved in IgE-mediated wheat allergy (Cabanillas 2019; Juhász et al. 2018). NCWS is a recently described wheat food-dependent distress, with an estimated prevalence of 0.16–13% (Brouns et al. 2019; Cabanillas 2019). The symptoms of NCWS may resemble those of CD, but do not involve damages to the intestine. There is no evidence for the involvement of autoimmunity or IgE-mediated reaction in NCWS; instead, activation of the innate immune system may play a role in this condition (Cabanillas 2019). The pathogenesis of NCWS is still poorly understood. It is likely that wheat ATIs and certain gluten protein components (e.g., gliadins) may be involved in triggering the disorder (Cabanillas 2019; Zevallos et al. 2017).

Therefore, a major challenge in wheat quality research is to enhance the end-use properties of grains while minimizing the immunogenic potential of gluten proteins (Altenbach 2017; Shewry and Tatham 2016; van den Broeck et al. 2009). To tackle this challenge effectively, a sound understanding of the expression, accumulation and genetic variation of gluten proteins is needed. Conventional methods, such as sodium dodecylsulphate–polyacrylamide gel electrophoresis (SDS-PAGE) and high-performance liquid chromatography (HPLC), although useful for characterizing HMW-GSs that have fewer members expressed in wheat, are insufficient for high resolution analysis of LMW-GSs and gliadins. This is because LMW-GSs and gliadins are generally expressed from multigene families with some members being highly similar in sequence, molecular size and expression profiles (Altenbach 2017; Shewry et al. 2003). This problem also complicates the matching of different LMW-GSs and gliadins accumulated in the grains to their corresponding genes and transcripts (Dupont et al. 2011). However, with the advent of structural and functional genomics and the availability of genomic information for wheat in recent years (IWGSC 2018; Uauy 2017), the difficulties outlined above are largely relieved, and our understanding on gluten protein expression profiles and functions has been substantially improved over the last 10 years. Consequently, the main objective of this review is to summarize the progress made in the genomic and functional genomics analyses of wheat gluten proteins. The prospect for simultaneously improving wheat end-use and health-related traits by genomic approaches will also be briefly discussed.

## Genomic analysis of gluten quality-related chromosomal loci and genes

Although it has long been known that the genes encoding glutenins and gliadins are carried in complex chromosomal loci (Shewry et al. 2003), only recently have systematic efforts been made to elucidate the organization of these loci by using the genome sequence information obtained for the hexaploid wheat variety Chinese Spring (CS) and closely related diploid and tetraploid wheat species (Avni et al. 2017; IWGSC 2018; Ling et al. 2018; Luo et al. 2017; Zhao et al. 2017). The homoeologous *Glu*-*1* loci (*Glu*-*A1*, -*B1* and -*D1*), carrying HMW-GS genes and located on the long arms of group 1 chromosomes, are relatively simple. Two paralogous HMW-GS genes, encoding one x and one y subunit, respectively, exist in each *Glu*-*1* locus, with the two paralogs separated by approximately 52–180 kb (Gu et al. 2006). The intergenic space of the two HMW-GS genes carries transposon elements as well as two genes predicted to encode a globulin and a protein kinase, respectively; immediately upstream of the x-type HMW-GS gene resides another globulin gene and a putative receptor kinase gene.

The three homoeologous composite loci carrying *Gli*-*1* and *Glu*-*3* (*Gli*-*A1/Glu*-*A3*, *Gli*-*B1/Glu*-*B3* and *Gli*-*D1/Glu*-*D3*), located on the short arms of group 1 chromosomes, are highly complex. Recent genomic studies in CS and the D genome donor species *Aegilops tauschii* (Aet) show clearly that *Gli*-*1* and *Glu*-*3* are physically linked, with *Gli*-*1* located upstream of *Glu*-*3* (Dong et al. 2016; Huo et al. 2018a). The precise physical size of a *Gli*-*1*/*Glu*-*3* composite locus is unknown at present, but is likely larger than 2 Mb. Based on the data gathered from CS and Aet, in each *Gli*-*1* region, there are a number of genes coding for γ-gliadins (4–5), δ-gliadins (1–2) or ω-gliadins (3–8). In each *Glu*-*3* region, there are 4–7 LMW-GS genes. There are also a few LMW-GS genes located outside of the main *Glu*-*3* region, probably resulting from translocation events. Another prominent feature shared by *Gli*-*1*/*Glu*-*3* composite loci is the presence of multiple copies of predicted receptor-like kinase genes and genes encoding the NLR proteins with nucleotide-binding domain and leucine-rich repeats. The genes specifying γ- or δ-gliadins are usually clustered together, so are those coding for ω-gliadins, but those encoding LMW-GSs are frequently separated by one or more *NLR* genes. In addition, a couple of syntenic ancestral genes are conserved among homoeologous *Gli*-*1*/*Glu*-*3* loci, which divide the genomic regions into four blocks, with blocks 1 and 2 encompassing *Gli*-*1* and blocks 3 and 4 covering *Glu*-*3*.

Genomic insight has also been gained into the three α-gliadin chromosomal loci (*Gli*-*A2*, -*B2* and -*D2*) located on the short arms of group 6 chromosomes (Huo et al. 2017, 2018b). In CS and Aet, the analyzed genomic regions carrying *Gli*-*A2*, -*B2* or -*D2* range from 387 to 836 kb, with the copy number of α-gliadin genes in the three loci varying from 12 to 24. *Gli*-*A2*, -*B2* or -*D2* regions are flanked by glutamate receptor-like (GRL) genes, with two *GRL* members at the 5′ end and one at the 3′ end; an internal insertion of another *GRL* member divides each *Gli*-*2* region into two subregions. Unlike *Gli*-*1*/*Glu*-*3* regions, *Gli*-*2* loci are less interrupted by non-prolamin genes. The structure of the *Gli*-*D2* locus in a Chinese wheat cultivar Xiaoyan 81 (Xy81) is similar but not identical to that present in CS and Aet. Several α-gliadin gene members present in CS and Aet are deleted in Xy81. However, two α-gliadin genes in Xy81 *Gli*-*D2* are each duplicated once, thus maintaining a total of 10 such genes (Li et al. 2018). These data demonstrate allelic variation of *Gli*-*D2* among different wheat materials, which may also happen to *Gli*-*A2* and -*B2*.

In addition to glutenins and gliadins, a number of studies have reported the expression of avenin-like proteins (ALPs) in wheat grains and increasing evidence on their influence of dough functionality. The transcripts for two types of ALPs (avenin-like a and avenin-like b) were originally discovered in the analysis of differentially expressed storage protein transcripts in *Aegilops* and wheat seeds (Kan et al. 2006). Type-a ALPs have a molecular mass of ~ 18 kDa and carry 14 conserved cysteine (cys) residues in their deduced proteins. On the other hand, type-b ALPs possess either 18 or 19 cys residues and have a molecular mass around 34 kDa. The two types of ALPs were renamed as farinins and purinins, respectively, by Kasarda et al. (2013). The larger molecular mass of type-b ALPs is mainly due to the duplication of an internal cys-rich domain of ~ 120 amino acids. Type-a ALPs are related to the LMW gliadins reported previously and may not be incorporated into the gluten polymers (Kasarda et al. 2013). In contrast, type-b ALPs have been detected in gluten polymers by both proteomic and transgenic studies (Kasarda et al. 2013; Ma et al. 2013a, b; Mamone et al. 2009; Vensel et al. 2014). The genes coding for ALPs are present in wheat and a wide range of Triticeae species (Chen et al. 2008, 2016; Kan et al. 2006). By searching the annotated genome sequence of CS, a total of 15 genes, six for type-a ALPs, six for type-b ALPs and another three for type-c ALPs, which represent a previously unrecognized class of ALPs, have been identified (Zhang et al. 2018a, b). These genes are located on chromosome arms 4AL, 7AS and 7DS, respectively, with five members (two for type-a, two for type-b and one for type-c ALPs) on each arm. Finally, evidence for the contribution of type-b ALPs to wheat dough functionality and end-use quality has been obtained by several studies (Chen et al. 2010, 2016; Ma et al. 2013a, b). Potential effects of other two types of ALPs on wheat gluten, dough and end-use properties remain to be determined.

The genomic organizations outlined above are obtained from only a limited number of genotypes. Variations to them are to be expected in wheat germplasm and closely related species, because the genomic regions carrying these highly complicated loci are subjected to independent and dynamic evolution (Gu et al. 2006; Huo et al. 2018a, b). Moreover, these loci are constantly modified by wheat breeding efforts because of their influences on end-use and health-related traits (Branlard et al. 2001; Dong et al. 2013, 2017; Gras et al. 2001; Wrigley et al. 2009).

## Genes regulating the expression of gluten proteins

Gluten proteins are specifically and primarily expressed in the endospermic tissues of developing wheat grains. Transcriptional regulation, brought about by intricate interactions between *cis*- and *trans*-acting factors, plays a key role in the control of gluten gene expression. There exist a large number of *cis*-elements in the promoter region of glutenin and gliadin genes. For example, the GCN4-like motif (GLM) and prolamin box (P-box), which are bound by basic leucine zipper (bZIP) and DNA binding with one finger (DOF) transcription factors (TFs), respectively, are present in the promoter regions of gluten genes, including HMW-GS, LMW-GS and α/β-gliadin genes (Albani et al. 1997; Dong et al. 2007; Juhász et al. 2011; Li et al. 2019; Noma et al. 2016; Ravel et al. 2014; She et al. 2011; Thomas and Flavell 1990; van Herpen et al. 2008b; Wang et al. 2013).

Two recent studies have provided substantial insights into the presence and function of conserved *cis*-regulatory modules (CCRM) in the promoters of HMW-GS genes (Li et al. 2019; Ravel et al. 2014). In the former study, wheat lines transformed with various promoter:GUS fusion constructs of a HMW-GS gene were developed and analyzed. The results showed that the 300 bp region, upstream of the translation initiation codon and carrying CCRM1 (− 300 to − 101), is sufficient for conferring endospermic expression of HMW-GS gene; the more upstream CCRMs, i.e., CCRM2 (− 650 to − 400) and CCRM3 (− 950 to − 750), enhance the expression of HMW-GS gene but have no effect on their expression specificity. More detailed analysis of the 300 bp basal promoter suggests that CCRM1-1 (− 208 to − 101) is indispensable for HMW-GS gene expression in the endosperm tissues, whereas CCRM1-2 (− 300 to − 209) is required for the timely onset of HMW-GS gene expression in the endosperm. The CCRMs provide a general and useful framework for further dissecting the functions of different *cis*-elements in the transcriptional regulation of HMW-GS genes. However, it is worth noting that homoeologous and paralogous HMW-GS, LMW-GS and α/β-gliadin genes often show indel polymorphisms in their promoter regions, which result in differences in the numbers and types of *cis*-elements contained (Geng et al. 2014; Juhász et al. 2011; Noma et al. 2016; Wang et al. 2013). Li et al. (2019) suggest that the CCRMs defined for HMW-GS gene promoters are not well conserved for LMW-GS and gliadin genes, indicating that differences exist in the *cis*-acting elements carried by the promoters of different types of gluten genes. This phenomenon was also observed by previous studies (Juhász et al. 2011; van Herpen et al. 2008b; Wang et al. 2013).

Considerable progress has also been made in the genomic analysis of *trans*-acting factors that affect gluten gene expression. In the study by Plessis et al. (2013), a diverse set of candidate genes encoding putative transcription factors (TFs), histones or chromatin modification proteins were found to significantly associated with the composition of glutenins and gliadins. Many of the associated genes are orthologs of the barley genes with demonstrated roles in regulating grain storage protein accumulation. A number of the associated TFs have been functionally confirmed to regulate the expression of glutenins and gliadins (Table 2). The bZIP TFs SPA and SHP have been shown to promote and repress the transcription of HMW-GS and LMW-GS genes, respectively (Albani et al. 1997; Boudet et al. 2019; Ravel et al. 2009). Wheat prolamin binding factor (WPBF), a DOF TF, has been found required for efficient expression of LMW-GSs and gliadins in the grains (Dong et al. 2007; Moehs et al. 2019; Ravel et al. 2006). Another DOF TF, PBF-D, binds P-box element in the promoters of the HMW-GS genes *Glu*-*1By8* and -*1Dx2*, and its overexpression can significantly increase the accumulation levels of HMW-GSs in the grains of transgenic wheat plants (Zhu et al. 2018). Guo et al. (2015) characterized a regulatory module consisted of the MYB TF TaGAMyb and the histone acetyltransferase TaGCN5, which regulates the expression of the HMW-GS gene *Glu*-*1Dy* by establishing a histone H3 acetylation pattern conducive to active gene transcription. Sun et al. (2017) identified TaFUSCA3, which is a B3 domain-containing TF and can transactivate the promoter of the HMW-GS gene *Glu*-*1Bx7* through binding to the *cis*-element RY repeat. Finally, the wheat DME gene encoding 5-methylcytosine DNA glycosylase is required for efficient expression of LMW-GS and gliadin genes by active demethylation of their promoters in developing wheat grains (Wen et al. 2012). Taken together, the available data suggest that transcription regulation of gluten genes involves complex interactions in between different *cis*- and *trans*-acting factors. Differences in these interactions may underlie variations in the expression patterns of individual gluten genes.

**Table 2:** List of *trans*-acting factors functionally identified to regulate gluten gene expression

Factor (module)	Protein	Demonstrated function	References
SPA	bZIP TF	Promoting the transcription of HMW-GS and LMW-GS genes	Albani et al. (1997) and Ravel et al. (2009)
SHP	bZIP TF	Repressing the transcription of HMW-GS and LMW-GS genes	Boudet et al. (2019)
WPBF	DOF TF	Required for efficient expression of LMW-GSs and gliadins	Dong et al. (2007), Moehs et al. (2019) and Ravel et al. (2006)
PBF-D	DOF TF	Enhancing the transcription of HMW-GS genes *Glu*-*1By8* and -*1Dx2*	Zhu et al. (2018)
TaGAMyb–TaGCN5	MYB TF—Histone acetyl transferase	Promoting the transcription of HMW-GS gene *Glu*-*1Dy*	Guo et al. (2015)
TaFUSCA3	B3 domain-containing TF	Activating the transcription of HMW-GS gene *Glu*-*1Bx7*	Sun et al. (2017)
DME	5-Methylcytosine DNA glycosylase	Required for efficient accumulation of LMW-GSs and gliadins	Wen et al. (2012)

## Genome-wide analysis of gluten gene transcription

Genome-wide analysis of gluten gene transcription can yield useful information on the expression profiles of different gluten gene members, which aids investigations of the functional importance of specific gluten proteins in the control of end-use and health-related traits. Genomic analysis of HMW-GS gene expression is straightforward because the number of genes involved is few and the homoeologous and paralogous members are relatively easy to differentiate. However, such analysis is quite difficult for LMW-GS and gliadin genes because of the presence of multiple homoeologs and paralogs, many of which have high sequence similarity, in wheat genome. To date, three main approaches have been used to investigate genome-wide expression patterns of gluten genes. The first approach is based on the identification and analysis of expression sequence tags (ESTs) coupled with quantitative PCR assay of specific gene members. Using this approach, Kawaura et al. (2005) reported the expression of 36 α/β-gliadin and 15 LMW-GS genes in CS developing grains. In another wheat cultivar Butte, the expression of 5 HMW-GS, 22 LMW-GS, 23 α-gliadin, 13 γ-gliadin and 7 ω-gliadin genes was detected in the grains through EST analysis (Altenbach et al. 2010; Dupont et al. 2011). EST analysis has also revealed remarkable variations in the relative expression levels of α-gliadins specified by homoeologous *Gli*-*2* loci among different wheat genotypes, which may facilitate the development of wheat lines with decreased content of α-gliadins (Salentijn et al. 2009). The second approach uses oligonucleotide array hybridization to investigate the genes expressed during wheat grain development (Wan et al. 2008). By this approach, the genes encoding TaALPs (see above) and a new class of gliadin genes, corresponding to the δ-gliadins described by Anderson et al. (2012), were identified (Kan et al. 2006; Wan et al. 2013).

In the third approach, next-generation sequencing technologies, including Illumina HiSeq and PacBio long-read sequencing platforms, are employed to analyze gluten gene transcription in wheat grains. Long-read transcriptome sequencing, which can encompass the coding region of most eukaryotic transcripts, facilitates the identification and differentiation of the transcripts of homoeologous and paralogous gluten genes. The high-coverage short transcriptome reads yielded by HiSeq sequencing are useful for correcting the base errors associated with long-read sequencing and for estimating the expression levels of individual gluten gene members. With this approach, Dong et al. (2015) identified the transcripts for 6 HMW-GS, 14 LMW-GS, 32 α/β-gliadin, 14 γ-gliadin and 6 ω-gliadin genes in the developing grains of Xy81, with intact open reading frame (ORF) found in 5 HMW-GS, 12 LMW-GS, 25 α/β-gliadin, 12 γ-gliadin (including 1 δ-gliadin) and 4 ω-gliadin gene members. In CS, the use of a similar approach identified the transcripts for 10 LMW-GS, 25 α/β-gliadin, 11 γ-gliadin, 2 δ-gliadin and 7 ω-gliadin genes in the grains (Huo et al. 2018a, b). In general, the transcript levels of the gluten genes differed widely (Huo et al. 2018a, b; Wang et al. 2017). For example, in the developing grains of CS examined at 20 days after anthesis, the transcript levels of 25 α-gliadin genes varied by as much as 206-folds based on the data of fragments per kilobase per million mapped reads; no transcript was detected for one α-gliadin gene (i.e., *α*-*B17*) despite it carried an intact ORF (Altenbach et al. 2019a, b). Together, the above studies show that the third approach is more powerful for transcriptomic analysis of gluten gene expression in wheat.

## Proteomic analysis of gluten proteins

Despite the fact that gluten gene expression is primarily regulated at the transcriptional level, proteomic information is essential for understanding (1) accumulation levels of individual gluten gene members in grains, (2) roles of specific gluten proteins in gluten and dough functionalities and (3) effects of environmental factors and cultivation measures on gluten protein accumulation and function (Altenbach 2017; Ribeiro et al. 2013). The basic steps in gluten proteomic studies include separation of gluten proteins by two-dimensional gel electrophoresis (2-DE), excision and enzymatic digestion of protein spots from 2-DE gels, and identification of proteins by various types of mass spectrometry (MS) methods, such as tandem MS (MS/MS), electrospray ionization tandem mass spectrometry (ESI/MS/MS) and matrix-assisted laser desorption/ionization time-of-flight mass spectrometry (MALDI-TOF–MS) (Dong et al. 2010; Dupont et al. 2011; Ferranti et al. 2007; Liu et al. 2010; Mamone et al. 2005, 2009; Muccilli et al. 2005). In addition to 2-DE, gel-free methods based on liquid chromatography (LC) have also been employed for separating protease-digested gluten proteins for MS analysis (Bromilow et al. 2017a; Colgrave et al. 2015; Fiedler et al. 2014; Schalk et al. 2017; Uvackova et al. 2013). Recently, Bromilow et al. (2017a) showed that LC–MS/MS analysis using a combination of QTOF (quadrupole time of flight) and LTQ (linear ion trap quadrupole) platforms is desirable for more comprehensive characterization of gluten proteins.

### Proteomic insight into gluten protein accumulation in wheat grains

Owing to the multiplicity of gluten proteins and high sequence similarities among some LMW-GS or gliadin protein members, it has been difficult to distinguish between closely related gluten protein homologs in proteomic experiments. This problem is further aggravated by the fact that the enzyme trypsin commonly used for digesting proteome samples does not have many cleavage sites in gluten proteins, which is due to the presence of the repetitive motifs rich in glutamine and proline and the low percentages of arginine and lysine residues required for trypsin digestion in these proteins (Dupont et al. 2011). However, these difficulties are largely overcome by digesting each gluten protein sample separately with multiple proteases (e.g., trypsin, thermolysin and chymotrypsin) (Altenbach et al. 2010; Dupont et al. 2011). A further problem in gluten proteomic studies is to match accumulated gluten proteins to their corresponding encoding genes. This effort is likewise complicated by the high copy of gluten genes and strong sequence similarities among some gluten gene members. To tackle this problem, it is necessary to develop and use cultivar-specific gluten gene sequence database (Altenbach et al. 2010; Bromilow et al. 2017b). For this purpose, earlier studies used gluten gene transcripts reconstructed from ESTs or the genomic sequences of specific gluten genes determined from sequencing bacterial artificial clones (Altenbach et al. 2010; Dong et al. 2010). Subsequently, full-length gluten gene transcripts identified from PacBio long-read RNA sequencing data were employed for the matching (Wang et al. 2017). Recently, Altenbach et al. (2019a, b) used the annotated reference genome sequence information for matching gluten proteins to their cognate genes in CS.

Through the various efforts outlined above, valuable proteomic information has been obtained for the gluten proteins of a number of wheat cultivars. In Butte, the expression of 5 HMW-GSs, 22 LMW-GSs, 23 α-gliadins, 13 γ-gliadins and 7 ω-gliadins in the flour was detected (Dupont et al. 2011). In Xiaoyan 54, the accumulation of 11 LMW-GSs in the grains was supported by both transcriptional and proteomic data (Dong et al. 2010). In Xy81, a combination of transcriptomic and proteomic analyses revealed the accumulation of 38 gliadins in mature grains, which included 21 α-, 11 γ-, 1 δ- and 5 ω-gliadins (Wang et al. 2017). Cho et al. (2018) identified the expression of 23 α-gliadins, 11 γ-gliadins and 5 ω-gliadins in the grains of the Korean cultivar Keumkang, although no attempt was made to link the gluten proteins to their coding genes. In CS, six HMW-GS genes including two pseudogene members were characterized, with the four active members expressing 1Bx7, 1By8, 1Dx2 and 1Dy12 subunits accumulated in the grains (van den Broeck et al. 2009). Through analyzing the reference genome sequencing data and conducting additional validation experiments, Huo et al. (2018a, b) identified a complete set of gliadin genes (including 47 for α-gliadin, 14 for γ-gliadin, 5 for δ-gliadin and 19 for ω-gliadins) and a total of 17 LMW-GS genes for CS; but the number of genes with intact ORF was found to be 26 for α-gliadins, 11 for γ-gliadins, 2 for δ-gliadins, 7 for ω-gliadins and 10 for LMW-GSs. Interestingly, proteomic analysis of CS flour samples identified the protein products for only 16 α-gliadin, 10 γ-gliadin, 1 δ-gliadin, 6 ω-gliadin and 9 LMW-GS proteins; for the gluten genes whose products were not found, they were either expressed at relatively low levels or their deduced products showed high similarities to other identified gluten members (Altenbach et al. 2019a, b). Wang et al. (2017) also failed to find protein products for the four α-gliadin genes with relatively low transcript levels in their analysis of Xy81 gluten proteins. On the other hand, alpha-D8 was the most highly accumulated α-gliadin in CS flour, although its transcript level was very low compared to those of highly transcribed α-gliadins in CS developing grains (Altenbach et al. 2019a, b). These findings suggest that the transcription and translation of gliadin genes are regulated in complex manners in wheat, which require further efforts to be fully elucidated.

From the data available, it seems that wheat cultivars may accumulate approximately 20–30 α-gliadins, 10–15 γ-gliadins, 1–3 δ-gliadins, 5–8 ω-gliadins, 10–15 LMW-GSs and 3–5 HMW-GSs in their grains. These rough estimates may help future proteomic and biochemical studies of the main gluten proteins in commercial wheat. Meanwhile, additional work is needed to investigate the composition and accumulation levels of gluten proteins in more diverse wheat germplasm materials. This is particularly relevant for α-gliadins because their coding genes are more numerous and there are large discrepancies in the reported numbers of α-gliadin genes in the same wheat material or among different wheat genotypes. For example, 47 α-gliadin genes were identified for CS based on analyzing the reference genome sequence (Huo et al. 2018b), but 90 such genes were detected for CS through sequencing PCR clones (Noma et al. 2016). Anderson et al. (1997) estimated 150 α-gliadin genes for the wheat cultivar Cheyenne based on Southern blot hybridization signals. Therefore, in future studies on the expression of α-gliadins, it is essential to determine the precise copy numbers of their genes in the wheat lines to be examined using more accurate genomic or molecular techniques, such as targeted sequence capture or droplet digital PCR (Altenbach et al. 2019a, b; Jouanin et al. 2019b). This information, coupled with high-throughput grain transcriptomic and proteomic data, may enable efficient elucidation of the mechanisms underpinning the transcriptional and translational regulations of α-gliadin genes in wheat.

It is important to point out that many wheat cultivars and germplasm lines accumulate rye secalins in the grains because of carrying the 1BL/1RS translocation chromosome (Graybosch 2001). The *Sec*-*1* locus of 1RS harbors the genes that encode γ- and ω-secalins, which resemble wheat γ-gliadins and ω-gliadins, respectively (Chai et al. 2005; Clarke et al. 1996). Although these secalins have been found to negatively affect gluten, dough and end-use properties by many genetic and rheological studies (Barbeau et al. 2003; Dhaliwal et al. 1990), the precise copy numbers of γ- and ω-secalin genes carried by *Sec*-*1* are still not well understood, neither is it clear how many distinct γ- and ω-secalins are accumulated in the grains of 1BL/1RS wheat varieties. Nevertheless, three studies have estimated the size of *Sec*-*1* to be at least 145 kb or 195 kb and contained 15 or 18 ω-secalin genes (Clarke et al. 1996; Li et al. 2016; Yamamoto and Mukai, 2005). The transcripts for 17 different ω-secalin genes were detected in the developing grains of the 1BL/1RS variety Shimai 15 by PCR cloning of cDNAs (Li et al. 2016). Four ω-secalin protein bands were detected in a SDS-PAGE analysis of 14 1BL/1RS cultivars (Chai et al. 2016a, b), and multiple secalin protein spots were found in 2-DE/MS studies of the wheat genotypes carrying 1BL/1RS (Blechl et al. 2016; Gobaa et al. 2007). These data should aid more detailed proteomic investigations of secalin expression in wheat grains in the future.

### Proteomic investigation of posttranslational modification of gluten proteins

Posttranslational modification (PTM) is an important issue frequently investigated in proteomic analysis of gluten proteins. However, there is still no strong evidence for extensive PTMs of gluten proteins in wheat grains or during dough processing. Nevertheless, several types of PTMs have been recorded for specific gluten proteins. In HMW-GSs, the 1By subunits (e.g., 1By8 and 1By9) have been shown to undergo two posttranslational cleavages at the C-terminal tail, resulting in two minor proteoforms visible on protein gels (Nunes-Miranda et al. 2017). The enzyme responsible for the cleavages may be an asparaginyl endopeptidase. The loss of C-terminal end, which includes a conserved cys residue involved in disulfide bond formation, may have a negative impact on the promotion of gluten and dough functionalities by 1By subunits (Nunes-Miranda et al. 2017). In LMW-GSs, PTM has been found to play a significant role in the processing of the N-terminal end of m- and s-type subunits (Dupont et al. 2011; Egidi et al. 2014). By combining transgenic and proteomic investigations, Egidi et al. (2014) proposed a model for the maturation of m- and s-type LMW-GSs. For the m-type subunits, the signal peptide (19 residues) is processed, followed by a further removal of the subsequent glutamine residue by an aminopeptidase cleavage, thus leading the mature proteins starting with METSCIF; For the s-type subunits, in addition to signal peptide processing and removal of glutamine residue, a third step, possibly mediated by an asparaginyl endopeptidase, is executed, which removed the MEN fragment, and generates the mature protein starting with SHIPGL. In gliadins, an asparaginyl endopeptidase-mediated cleavage has been found involved in the processing of the ω-gliadins specified by *Gli*-*A1* or -*D1* loci (Dupont et al. 2004). Such a cleavage has also been proposed to occur in the processing of some farinin proteins (Kasarda et al. 2013). Finally, widespread glutamine deamidation in different types of gluten proteins has been revealed by several proteomic studies, although it is currently unclear if this modification occurs genuinely in the grains or caused by sample treatment during proteomic analysis (Bromilow et al. 2017a; Dupont et al. 2011; Martínez-Esteso et al. 2016). Phosphorylation, a common form of PTM, is not found for gluten proteins, although it is readily detected in many wheat grain proteins involved in diverse physiological and biochemical processes (Ma et al. 2014; Zhang et al. 2014; Zhen et al. 2017).

### Composition of gluten polymers as investigated using proteomic analysis

As pointed earlier, the quantity and size distribution of GMPs are important indicators of dough and end-use properties, and GMPs are quantitatively and functionally correlated with the amount of UPP complexes (see above). As demonstrated by Vensel et al. (2014), proteomic analysis represents an efficient tool for investigating the composition of UPP in wheat. In their study, the composition of a major UPP fraction (UPP peak 1), which was insoluble in 0.5% SDS and hence contained GMPs, was analyzed using 2-DE-MS/MS. HMW-GSs and LMW-GSs were found to be the main components, accounting for 28.52% and 44.72% of the fraction, respectively. The α-, γ- and ω-gliadins with an odd number of cys residue, and thus acting as glutenin chain terminators, were also identified (5.43%). In addition, this fraction contained monomeric gliadins (12.61%), serpins (3.41%), triticins (3.84%) and globulins (0.57%), which together made up 20.43% of the fraction. In parallel, the same study analyzed protein composition of the major extractable polymeric protein (EPP) fraction (EPP peak 1), which was soluble in 0.5% SDS and presumably contained smaller gluten polymers. HMW-GSs, chain terminating gliadins, serpins, triticins and globulins were also present in EPP, but their proportions were 20.18%, 14.67%, 5.74%, 7.21% and 2.55%, respectively. These data suggest that glutenins (HMW-GSs and LMW-GSs) are the dominant component in UPP. In contrast, EPP contains a decreased amount of HMW-GSs, but increased quantities of chain-terminating gliadins, serpins, triticins and globulins. Mueller et al. (2016) prepared and analyzed GMP gel, which is formed by the largest gluten polymers (with the molecular mass ranging from 5 to 20 million Da). They found that the major protein component in GMP gel is glutenins (~ 90%), with gliadins occupying only ~ 10%. No albumins or globulins were found in the GMP gel analyzed. From the information available, it seems that the larger the size of the gluten polymers the higher proportion of glutenins they contain, with gliadins and other proteins decreased accordingly. However, it is well known that the amount and size distribution of gluten polymers are affected by both genotypes and environments (Johansson et al. 2013; Ni et al. 2014; Zhao et al. 2011; Zhang et al. 2016). Therefore, further proteomic work using more wheat varieties cultivated in different environments is needed in order to obtain a better understanding of the dynamic changes in the composition of gluten polymers in response to genetic background and growth conditions.

### External factors on gluten protein accumulation

External changes, caused by fluctuations of environmental factors or application of cultivation measures, can have large effects on the performance and stability of end-use quality, which is mediated, at least partly, by altered expression, accumulation and function of gluten proteins (Altenbach 2012, 2017). Proteomic analysis provides an effective tool for deciphering the changes of wheat grain proteome (including gluten proteins) induced by external factors at a genome-wide level, and the findings have been reviewed in depth by Altenbach (2017). From the data reported so far, it appears that abiotic stresses generally induce complex proteomic changes in wheat grains, including decreased expression of the proteins and pathways involved in normal growth and physiological processes but up-regulated expression and function of those required for stress adaptation and tolerance, accompanied by significant reductions in graining filling period and kernel weight. Heat, drought or salt stress applied during flowering or post anthesis tend to increase the accumulation of α- and ω-gliadins and HMW-GSs, but exhibit differential effects on the accumulation of different LMW-GSs, with genotypes, types of stresses and growth stages when stress was encountered having significant influences on the changes of gluten proteins (Hurkman et al. 2013; Zhang et al. 2018a, b; Zhou et al. 2018; Yang et al. 2011). The expression of α-gliadins seems to be more strongly affected by heat and drought, indicating that the regulation of these proteins is more sensitive to abiotic stresses. Rebalancing of gluten protein accumulation often occurs, with the amount increased for some members but decreased for the others. These changes can lead to elevation of grain protein content (GPC), resulting in improvement of end-use quality-related parameters under stress conditions.

Changes in fertilizer application likewise trigger complex alterations in wheat grain proteome, with the effects varying according to the types and amounts of fertilizers applied and the timing of application (Altenbach et al. 2011; Altenbach 2017; Xue et al. 2019; Zörb et al. 2018). Appropriate application of nitrogen can significantly promote gluten protein accumulation and end-use quality. For example, nitrogen applied at the booting stage increases GPC and the contents of most gluten proteins, especially HMW-GSs and α- and ω-gliadins, which is accompanied by the formation of more and larger protein bodies and enhanced expression of several protein disulfide isomerases required for efficient GMP formation (Xue et al. 2016, 2019; Yu et al. 2017; Zhong et al. 2019). High nitrogen application also elevates GPC and accumulation levels of many gluten proteins, although it may lead to reduced nitrogen use efficiency (Zhen et al. 2018; Zheng et al. 2018; Zörb et al. 2018). Interestingly, Roy et al. (2019) demonstrated that restoring the expression of the HMW-GS gene *Glu*-*1Ay*, which is normally silenced in worldwide common wheat varieties, leads to increased GPC and breadmaking quality in the Australian variety Lincoln, indicating a new way of enhancing grain N accumulation and nitrogen use efficiency by enlarging the number of expressed HMW-GSs in wheat. Furthermore, a breakthrough in dissecting the molecular mechanism underlying nitrogen promotion of wheat GPC and GMPs was reported recently, which shows that adequate nitrogen supply enhances the availability of glutamine for different biological processes during grain development, and in the meantime, elevates GMPs by up-regulating the function of peptidyl-prolyl *cis*–*trans* isomerase (PPIase) through SUMOylation of PPIase with the aid of the small ubiquitin-related modifier 1 (Yu et al. 2018).

Lastly, sufficient availability of sulfur element in the soil has been shown to facilitate the synthesis of gluten proteins, particularly the S-rich α- and γ-gliadins and LMW-GSs (Bonnot et al. 2017; Grove et al. 2009; Zörb et al. 2010). Applications of phosphorous, magnesium, zinc and manganese fertilizers have also been found to confer beneficial effects on gluten protein synthesis in wheat grains (Gaj et al. 2013), although the specific proteomic changes involved remain to be determined.

## Genome-wide analysis of wheat sensitivity-related gluten proteins

Before the availability of wheat reference genome sequence, studies of wheat sensitivity-related gluten proteins and epitopes were largely limited to individual, or specific type(s), of gluten members (Gilissen et al. 2014; Shan et al. 2002; Tye-Din et al. 2010; van den Broeck et al. 2009; van Herpen et al. 2006). By analyzing the whole genome sequence of CS, Juhász et al. (2018) recently mapped and experimentally tested wheat immunoresponsive proteins at a genome-wide level. From the available studies, several main themes regarding CD and WDEIA have become apparent, with genome-wide information beginning to emerge for other wheat sensitivities (barker’s asthma and NCWS).

First, the T cell CD epitopes are present in all major families of gluten proteins (HMW-GSs, LMW-GSs and gliadins), with the most important ones detected in the α- and ω-gliadins encoded by wheat D subgenome. Juhász et al. (2018) identified 12 α- and ω-gliadins with comparatively high immune response, which were all encoded by D subgenome. The highly toxic CD epitopes carried by *Gli*-*D2* encoded α-gliadins are located in a 33-mer gliadin peptide resistant to the digestion by human protease (Shan et al. 2002). However, the number of gliadins with the 33-mer peptide is quite limited in number. In both CS and Xy81, only two α-gliadins were found to possess the 33-mer peptide among the diverse ranges of gliadins analyzed (Juhász et al. 2018; Li et al. 2018). The gluten proteins specified by B subgenome tend to carry fewer CD epitopes with weaker immunogenic potential (Juhász et al. 2018).

Second, CD epitopes are primarily located in the repetitive region of gluten proteins; the C-terminal domain can also carry CD epitopes but with weaker immunogenic potential (Juhász et al. 2018; Shewry 2019). For many gluten proteins, especially γ-gliadins, *Gli*-*D2* α-gliadins and the ω-gliadins encoded by *Gli*-*D1*, there usually exist multiple CD epitopes in their repetitive domain.

Third, a considerable proportion of gluten proteins do not carry, or have very few of, the CD epitopes known to date, particularly the α-gliadins encoded by wheat B subgenome. In CS, at least 10 α-gliadins do not carry CD epitopes, 9 of which are encoded by B subgenome (*Gli*-*B2*) (Huo et al. 2018b). Among the 38 α-, γ-, δ- and ω-gliadins found to accumulate in Xy81 grains, 10 members do not carry CD epitopes, which include 7 α-gliadins encoded by *Gli*-*B2*, 1 α-gliadin by *Gli*-*D2*, 1 ω-gliadin by *Gli*-*B1* and 1 δ-gliadin by *Gli*-*D1*; 8 members, including 6 encoded by *Gli*-*A2* and 2 by *Gli*-*B2*, have only 1 or 2 CD epitopes in their proteins (Wang et al. 2017). In wheat, the α-gliadins that do not carry or possess only 1 to 2, CD epitopes generally carry the CSTT motif; the great majority of CSTT gliadins are encoded by B subgenome (11 in CS and 10 in Xy81), with 1 or 2 specified by A or D subgenome (Huo et al. 2018b; Wang et al. 2017).

Fourth, the ω-5 gliadins encoded by *Gli*-*B1* locus and HMW-GSs are the main contributors to WDEIA (Juhász et al. 2018; Scherf et al. 2016). The WDEIA epitopes, QQFPQQQ, QQIPQQQ, QQSPQQQ and QQSPEQQ in ω-5 gliadins and QQPGQ, QQPGQGQQ and QQSGQGQ in HMW-GSs, come from the repetitive regions of the implicated gluten proteins. In addition, a recent study showed that wheat *Gli*-*D1* locus could also express the ω-5 gliadins highly reactive with the IgE antibody of WDEIA patients (Altenbach et al. 2018).

Fifth, no strong evidence has been obtained for the involvement of glutenins and gliadins in the elicitation of NCWS (Brouns et al. 2019; Cabanillas 2019), although there is evidence that consumption of low-gliadin bread confers beneficial changes to gut microbiota of NCWS patients (García-Molina et al. 2019). However, certain subclasses of ATIs are emerging as contributors to NCWS (Zevallos et al. 2017). The ATIs and many nsLTPs also contain the epitopes associated with baker’s asthma (Juhász et al. 2018).

Finally, the allergenic potential of gluten proteins is significantly affected by the growth environment. This is understandable considering that the expression of gluten proteins is frequently modulated by environmental factors and cultivation measures (see above). From the information available (Altenbach 2017; Juhász et al. 2018), it appears that high temperature stress increases the levels of CD epitopes owing to stimulation of α- and ω-gliadin accumulation, particularly the α-gliadins carrying the 33-mer peptide, while low temperature stress decreases the level of CD epitopes but increases the amount of certain immunostimulatory factors associated with WDEIA or baker’s asthma. Brzozowski and Stasiewicz (2017) found that water stress at the flowering stage increased the levels of immunostimulatory ω-gliadins. Boukid et al. (2017) reported that the level of toxic CD epitopes was affected by complex interactions among wheat cultivars, growth season’s climate conditions and breeding histories of the examined wheat varietal population. Therefore, a comprehensive understanding of the effects of growth conditions on the immunogenic potential of gluten proteins may come from the studies involving more diverse wheat genotypes cultivated in different environments.

## Prospect for concurrent improvement of wheat end-use and health-related traits

Although it is highly desirable to simultaneously improve grain end-use and health-related traits, the task is very challenging because many of the gluten proteins involved in wheat sensitivities are actually important participants of wheat end-use quality control. However, there are encouraging findings: a number of studies have shown that decreasing gliadin accumulation can reduce wheat sensitivity-related epitopes without affecting wheat end-use quality, and many gluten proteins do not carry the known wheat sensitivity-related epitopes. Thus, several strategies have been explored to identify wheat genotypes with reduced gluten protein accumulation or to create genetically modified lines with decreased gluten content (Rustgi et al. 2019). The following is a brief summary of the five main approaches that are promising for simultaneously improving wheat grain end-use and health-related traits through removing (or modifying) the toxic gluten proteins while enhancing the functions of the gluten members without disease epitopes (Table 3).

**Table 3:** Approaches used for decreasing immunogenic potential of wheat gluten proteins

Approach	Gluten proteins targeted	Main finding	References
RNA interference of gliadin genes	γ-Gliadins	Reduced expression of γ-gliadins in nine transgenic lines, accompanied by increased levels of α- and ω-gliadins; higher SDS-sedimentation values observed in six transgenic lines.	Gil-Humanes et al. (2008) and Pistón et al. (2011)
	α-, ω- and/or γ-gliadins	Gliadin expression strongly down-regulated (by 85.6% on average); transgenic wheat lines with very low levels of toxicity for CD patients obtained; many of the transgenic lines exhibiting improved end-use quality and nutritional value	Gil-Humanes et al. (2010, 2012, 2014a, b)
	ω-5 gliadins	Content of ω-5 gliadin decreased by 80% in one line and eliminated in another line; reactivity to the IgE antibody of WDEIA patients greatly reduced; dough functionality parameters improved	Altenbach and Allen (2011) and Altenbach et al. (2014a, b, 2015)
	α-Gliadins	Content of α-gliadin strongly reduced in two transgenic lines, compensated by increased levels of γ- and ω-gliadins, HMW-GSs and other seed proteins; no significant effect on flour functionality observed	Becker et al. 2012
	α-, γ- and ω-gliadins, LMW-GSs	Strong silencing of different types of gliadins and LMW-GSs achieved; several lines devoid of CD epitopes from the highly immunogenic α- and ω-gliadins; total protein and starch contents unaffected in the grains of the transgenic lines	Barro et al. (2016)
	Secalins in 1BL/1RS wheat line	Significant decreases in multiple secalins and closely related ω-gliadins; increased levels of α-gliadins and HMW-GSs; improved dough functionality for two transgenic lines	Blechl et al. (2016)
	ω-1,2 gliadins	Effective silencing of ω-1,2 gliadins achieved; immunoreactivity of flour protein to the antibodies of CD patients decreased; dough functionality improved in one transgenic line	Altenbach et al. (2019a, b)
Deletion of gluten chromosomal loci	α-, γ- and ω-gliadins	Three deletion lines with null allele at *Gli*-*D1*, *Gli*-*B1* or *Gli*-*B2* developed; immunoreactivity of flour proteins reduced by 6–18% in the deletion lines	Waga et al. (2013)
	ω-1,2 and ω-5 gliadins	Wheat genotypes lacking both ω-1,2 and ω-5 gliadins developed, with approximately 30% reduction of gliadin immunoreactivity but improved gluten functionality	Waga and Skoczowski (2014)
	α-Gliadins	Three deletion lines lacking *Gli*-*A2*, *Gli*-*D2* or *Gli*-*A2*/*Gli*-*D2* prepared; large decreases in α-gliadin expression observed in the three lines; the 33-mer peptide bearing gliadins not detected in the lines missing *Gli*-*D2* or *Gli*-*A2*/*Gli*-*D2*	Camerlengo et al. (2017)
	α-Gliadins	Six deletion lines lacking gliadin chromosome loci developed; the line missing *Gli*-*D2* showed improved dough functionality and breadmaking quality, with the level of CD epitopes significantly decreased	Wang et al. (2017) and Li et al. (2018)
Expression of engineered glutenases	α-Gliadins	Transgenic wheat expressing engineered glutenases developed, which could degrade the CD epitopes carried by α-gliadins under simulated gastrointestinal conditions	Osorio et al. (2019)
Manipulation of regulators	LMW-GSs and gliadins	Expression of gliadins and LMW-GSs in wheat grains significantly decreased by suppressing the function of 5-methylcytosine DNA glycosylase or the WPBF TF	Wen et al. (2012) and Moehs et al. (2019)
Genome editing of gliadin genes	α-Gliadins	Effective mutation of multiple α-gliadin genes accomplished using CRISPR/Cas9 mediated genome editing, with the immunoreactivity of gluten proteins reduced by up to 85%	Sánchez-León et al. (2018) and Jouanin et al. (2019a)

The first approach is to use RNA interference (RNAi) to silence the expression of all, or specific types of, gliadins in transgenic wheat plants (Table 3). In general, the gliadin silenced lines showed decreased gliadin content, lowered immunogenic potential and improved end-use quality parameters (Becker et al. 2012; Gil-Humanes et al. 2008, 2010, 2012, 2014a, b; Pistón et al. 2011; Zörb et al. 2013). For example, Barro et al. (2016) could concurrently silence the expression of α-, γ- and ω-gliadins using combinations of RNAi constructs, which eliminated CD epitopes from the highly immunogenic α- and ω-gliadins but without affecting total protein and starch contents in the grains. RNAi has been also used successfully to silence the expression of ω-5 gliadins; the resultant lines exhibited improved flour quality and may be useful for decreasing the incidence of WDEIA (Altenbach and Allen 2011; Altenbach et al. 2014a, b, 2015). Recently, Altenbach et al. (2019a, b) decreased the expression of ω-1,2 gliadins by RNAi, with the resulting lines showing significantly reduced immunogenic potential and substantially improved end-use quality parameters. The rye secalins expressed in wheat background due to the presence of 1BL/1RS translocation chromosome can be effectively silenced using RNAi, with the transgenic lines showing enhanced dough functional properties (Blechl et al. 2016; Chai et al. 2016a, b). Lastly, Gil-Humanes et al. (2014b) revealed that the low-gliadin transgenic wheat lines produced using RNAi had also improved nutritional quality because of increased lysine content in the grains; García-Molina et al. (2019) noted that consumption of the bread made with the flour of a low-gliadin wheat (E82), which showed 98.1% reduction of gluten content when evaluated using the R5 antibody, induced positive changes in the composition of gut microbiota in NCWS patients.

The second approach is to develop wheat deletion lines lacking one or more gliadin chromosome loci (Table 3). Waga et al. (2013) developed and assessed three gliadin deletion lines with null allele at *Gli*-*D1*, *Gli*-*B1* or *Gli*-*B2* and found that the immunoreactivity of flour proteins of the deletion lines was 6–18% lower than that of wild-type control. Subsequently, wheat genotypes lacking both ω-1,2 and ω-5 gliadins were developed, which had a 30% decrease in gliadin immunoreactivity but improved gluten content and strength (Waga and Skoczowski 2014). Similarly, Camerlengo et al. (2017) described three wheat deletion lines lacking *Gli*-*A2*, *Gli*-*D2* and *Gli*-*A2*/*Gli*-*D2*, respectively. These mutant lines had large decreases in α-gliadin expression, with the 33-mer peptide bearing gliadins not detected in the ones missing *Gli*-*D2* or *Gli*-*A2*/*Gli*-*D2*. Wang et al. (2017) reported the development of six wheat deletion lines each lacking one of the six gliadin chromosome loci. The line DLGliD2, which has *Gli*-*D2* deleted, showed improved dough functionality and breadmaking quality, with the level of CD epitopes significantly decreased (Li et al. 2018; Wang et al. 2017). Being non-transgenic, these deletion lines may be directly used in developing the wheat lines less toxic to the individuals affected by wheat sensitivity problem.

The third approach is to develop transgenic wheat lines expressing engineered “glutenases” for targeted degradation of celiac inducing epitopes in the intestine (Table 3). Osorio et al. (2019) created transgenic wheat lines with endosperm specific expression of barley endoprotease B2 (EP-HvB2), *Flavobacterium meningosepticum* prolyl endopeptidase (PE-FmPep) and *Pyrococcus furiosus* prolyl endopeptidase (PE-PfuPep). These preconditioned gluten detoxifiers (EP-HvB2 + PE-FmPep or EP-HvB2 + PE-PfuPep) did not affect the end-use quality of flour, but could degrade the CD epitopes contained in the 33-mer gliadin peptide under simulated gastrointestinal conditions. Up to 72% reduction in the immunogenic peptides was found for the transgenic lines, thus opening the possibility of developing an intraluminal enzyme therapy for CD without negatively affecting wheat end-use quality and overall agronomical performance.

The fourth approach is to decrease the expression of gluten proteins through manipulating the regulators controlling prolamin gene expression (Table 3). Wen et al. (2012) demonstrated that functional suppression of wheat *DME* gene, which encodes 5-methylcytosine DNA glycosylase, led to decreased accumulation of LMW-GSs and gliadins. Recently, Moehs et al. (2019) showed that elimination of the homoeologous genes encoding WPBF resulted in decreased accumulation of LMW-GSs and gliadins, which together accounted for 50–60% of wheat gluten proteins. These regulatory genes are potentially useful targets for developing low-gluten wheat lines, although efforts are needed to alleviate the side effects on agronomic traits associated the mutation of these genes.

The fifth approach is to modify gluten gene expression using genome editing (Table 3). Genome editing is a rapidly developing technology for introducing site targeted mutations to genic and regulatory regions (Knott and Doudna 2018; Yin et al. 2017). It consists of a nuclease (e.g., Cas9 and Cpf1) and a single guide RNA (sgRNA); the sgRNA is complementary to the target site, which binds to the nuclease and then directs the ribonuclear protein complex to the specific target site. Depending on the methods used, the editing results in either indel mutations or base changes (i.e., A to G or C to T) at the target site (Chen et al. 2019). Genome editing can be performed for single or multiple genes with one or more sgRNAs. Using CRISPR/Cas9 mediated genome editing, Sánchez-León et al. (2018) succeeded in mutating a large number of α-gliadin genes in wheat (up to 35), with the immunoreactivity of gluten proteins reduced by as much as 85%. Jouanin et al. (2019a) confirmed that CRISPR/Cas9 is effective in mutating α-gliadin genes and further showed this method could be used to mutate γ-gliadin genes in wheat.

Of the different approaches outlined above, genome editing is relatively new, and its utility in modifying gluten protein expression remains to be fully exploited. In addition to mutations created by indel-inducing CRISPR/Cas9, as demonstrated by Sánchez-León et al. (2018), various types of base editors, as reviewed recently (Chen et al. 2019; Mishra et al. 2019), may be employed to correct the gluten proteins that are important in end-use quality control but harbor wheat sensitivity-related epitopes. The versatility of genome editing took a big step forward recently with the development of prime editing, which can engineer all 12 forms of base substitutions, insertions (1 to ≥ 44 bp), deletions (1 to ≥ 80 bp) and combinations of the different types of alterations in a predetermined target site (Anzalone et al. 2019). The ability to conduct multiplex editing makes it possible to modify different families and subtypes of gluten genes in a high throughput manner. Lastly, the different genome editing methods may also be useful for enhancing the end-use quality controlling function of those epitope-free gluten members. These attributes, plus the ease to obtain genome-edited but transgene-free wheat plants (Sánchez-León et al. 2018), support the idea that genome editing has the highest potential in refining gluten protein composition for concurrent improvement of wheat end-use and health-related traits.

## Conclusion

Genomic and functional genomics studies have substantially improved the understanding on gluten chromosomal loci and genes and the mechanisms regulating gluten protein expression. It is now possible to elucidate the complete repertoire of gluten genes and proteins in wheat and to monitor their expression changes at whole genome level in response to alterations in the growth environments. Furthermore, an important molecular clue underlying the promotion of gluten protein functionality by favorable environmental factor, i.e., enhancement of GMPs by SUMOylation of PPIase under adequate nitrogen conditions, has emerged. Meanwhile, genome-wide insights have been gained into the types and structures of immunogenic gluten proteins. Valuable approaches have been tested for simultaneously improving wheat end-use and health-related traits. However, there are still important gaps in the knowledge on the molecular networks controlling gluten gene expression and on the biochemical and biophysical mechanisms underlying gluten protein interactions. In addition, more efforts are needed to grasp, and to make efficient use of, the large genetic variations in gluten protein structure and expression in wheat germplasm. Looking into the future, the combination of genomic, functional genomics and genome editing studies will speed up the basic and applied research on gluten proteins, thus enabling efficient development of elite wheat varieties with the end-use and health-related traits desired by different consumption needs.

## References

[CR1] Albani D, Hammond-Kosack M, Smith C, Conlan S, Colot V, Holdsworth M (1997). The wheat transcriptional activator SPA: a seed-specific bZIP protein that recognizes the GCN4-like motif in the bifactorial endosperm box of prolamin genes. Plant Cell.

[CR2] Altenbach SB (2012). New insights into the effects of high temperature, drought and post-anthesis fertilizer on wheat grain development. J Cereal Sci.

[CR3] Altenbach SB, Colgrave ML (2017). Proteomics of wheat flour. Proteomics in food science.

[CR4] Altenbach SB, Allen PV (2011). Transformation of the US bread wheat ‘Butte 86’ and silencing of omega-5 gliadin genes. GM Crops.

[CR5] Altenbach SB, Vensel WH, Dupont FM (2010). Integration of transcriptomic and proteomic data from a single wheat cultivar provides new tools for understanding the roles of individual alpha gliadin proteins in flour quality and celiac disease. J Cereal Sci.

[CR6] Altenbach SB, Tanaka CK, Hurkman WJ, Whitehand LC, Vensel WH, Dupont FM (2011). Differential effects of a post-anthesis fertilizer regimen on the wheat flour proteome determined by quantitative 2-DE. Proteome Sci.

[CR7] Altenbach SB, Tanaka CK, Allen PV (2014). Quantitative proteomic analysis of wheat grain proteins reveals differential effects of silencing of omega-5 gliadin genes in transgenic lines. J Cereal Sci.

[CR8] Altenbach SB, Tanaka CK, Seabourn BW (2014). Silencing of omega-5 gliadins in transgenic wheat eliminates a major source of environmental variability and improves dough mixing properties of flour. BMC Plant Biol.

[CR9] Altenbach SB, Tanaka CK, Pineau F, Lupi R, Drouet M, Beaudouin E (2015). Assessment of the allergenic potential of transgenic wheat (*Triticum aestivum*) with reduced levels of ω5-gliadins, the major sensitizing allergen in wheat-dependent exercise-induced anaphylaxis. J Agric Food Chem.

[CR10] Altenbach SB, Chang HC, Simon-Buss A, Jang Y-R, Denery-Papini S, Pineau F (2018). Towards reducing the immunogenic potential of wheat flour: omega gliadins encoded by the D genome of hexaploid wheat may also harbor epitopes for the serious food allergy WDEIA. BMC Plant Biol.

[CR11] Altenbach SB, Chang HC, Simon-Buss A, Mohr T, Huo N, Gu YQ (2019). Exploiting the reference genome sequence of hexaploid wheat: a proteomic study of flour proteins from the cultivar Chinese Spring. Func Integr Genomics.

[CR12] Altenbach SB, Chang HC, Yu XB, Seabourn BW, Green PH, Alaedini A (2019). Elimination of omega-1,2 gliadins from bread wheat (*Triticum aestivum*) flour: effects on immunogenic potential and end-use Quality. Front Plant Sci.

[CR13] Anderson OD, Litts JC, Greene FC (1997). The α-gliadin gene family. I. Characterization of ten new wheat α-gliadin genomic clones, evidence for limited sequence conservation of flanking DNA, and Southern analysis of gene family. Theor Appl Genet.

[CR14] Anderson OD, Dong L, Huo N, Gu YQ (2012). A new class of wheat gliadin genes and proteins. PLoS ONE.

[CR15] Anzalone A, Randolph PB, Davies JR, Sousa AA, Koblan LW, Levy JM (2019). Search- and -replace genome editing without double-strand breaks or donor DNA. Nature.

[CR16] Avni R, Nave M, Barad O, Baruch K, Twardziok SO, Gundlach H (2017). Wild emmer genome architecture and diversity elucidate wheat evolution and domestication. Science.

[CR17] Bangur R, Batey IL, McKenzie E, MacRitchie F (1997). Dependence of extensograph parameters on wheat protein composition measured by SE-HPLC. J Cereal Sci.

[CR18] Barbeau WE, Schwarzlaff SS, Uriyo MG, Johnson JM, Harris CH, Griffey CA (2003). Origin and practical significance of the sticky dough factor in 1BL/1RS wheats. J Sci Food Agric.

[CR19] Barro F, Iehisa JC, Giménez MJ, García-Molina MD, Ozuna CV, Comino I (2016). Targeting of prolamins by RNAi in bread wheat: effectiveness of seven silencing-fragment combinations for obtaining lines devoid of coeliac disease epitopes from highly immunogenic gliadins. Plant Biotechnol J.

[CR20] Becker D, Wieser H, Koehler P, Folck A, Mühling KH, Zörb C (2012). Protein composition and techno-functional properties of transgenic wheat with reduced α-gliadin content obtained by RNA interference. J Appl Bot Food Qual.

[CR21] Blechl A, Beecher B, Vensel W, Tanaka C, Altenbach S (2016). RNA interference targeting rye secalins alters flour protein composition in a wheat variety carrying 1BL.1RS translocation. J Cereal Sci.

[CR22] Bonnot T, Bancel E, Alvarez D, Davanture M, Boudet J, Pailloux M (2017). Grain subproteome responses to nitrogen and sulfur supply in diploid wheat *Triticum monococcum ssp. monococcum*. Plant J.

[CR23] Boudet J, Merlino M, Plessis A, Gaudin JC, Dardevet M, Perrochon S (2019). The bZIP transcription factor SPA heterodimerizing protein represses glutenin synthesis in *Triticum aestivum*. Plant J.

[CR24] Boukid F, Prandi B, Sforza S, Sayar R, Seo YW, Mejri M (2017). Understanding the effects of genotype, growing year, and breeding on Tunisian durum wheat allergenicity. 2. The celiac disease case. J Agric Food Chem.

[CR25] Branlard G, Dardevet M, Saccomano R, Lagoutte F, Gourdon J (2001). Genetic diversity of wheat storage proteins and bread wheat quality. Euphytica.

[CR26] Bromilow SN, Gethings LA, Langridge JI, Shewry PR, Buckley M, Bromley MJ (2017). Comprehensive proteomic profiling of wheat gluten using a combination of data-independent and data-dependent acquisition. Front Plant Sci.

[CR27] Bromilow S, Gethings LA, Buckley M, Bromley M, Shewry PR, Langridge JI (2017). A curated gluten protein sequence database to support development of proteomics methods for determination of gluten in gluten-free foods. J Proteom.

[CR28] Brouns F, van Rooy G, Shewry P, Rustgi S, Jonkers D (2019). Adverse reactions to wheat or wheat components. Compr Rev Food Sci F.

[CR29] Brzozowski B, Stasiewicz K (2017). Effects of water stress on the composition and immunoreactive properties of gliadins from two wheat cultivars: Nawra and Tonacia. J Sci Food Agric.

[CR30] Cabanillas B (2019). Gluten-related disorders: celiac disease, wheat allergy, and nonceliac gluten sensitivity. Crit Rev Food Sci Nutr.

[CR31] Camerlengo F, Sestili F, Silvestri M, Colaprico G, Margiotta B, Ruggeri R (2017). Production and molecular characterization of bread wheat lines with reduced amount of α-type gliadins. BMC Plant Biol.

[CR32] Chai JF, Lu X, Jia JZ (2005). Homoeologous cloning of ω-secalin gene family in a wheat 1BL/1RS translocation. Cell Res.

[CR33] Chai JF, Zhang CM, Ma XY, Wang HB (2016). Molecular identification of ω-secalin gene expression activity in a wheat 1B/1R translocation cultivar. J Integr Agric.

[CR34] Chai JF, Wang HB, Ma XY, Zhang CM, Dong FS (2016). Effect of ω-secalin gene silencing on processing quality of wheat 1B/1R translocation line. Acta Agron Sin.

[CR35] Chen P, Wang CD, Li KX, Chang JL, Wang YS, Yang G (2008). Cloning, expression and characterization of novel avenin-like genes in wheat and related species. J Cereal Sci.

[CR36] Chen P, Li R, Zhou R, He GY, Shewry PR (2010). Heterologous expression and dough mixing studies of a novel cysteine-rich avenin-like protein. Cereal Res Commun.

[CR37] Chen XY, Cao XY, Zhang YJ, Islam S, Zhang JJ, Yang RC (2016). Genetic characterization of cysteine-rich type-b avenin-like protein coding genes in common wheat. Sci Rep.

[CR38] Chen K, Wang Y, Zhang R, Zhang H, Gao C (2019). CRISPR/Cas Genome editing and precision plant breeding in agriculture. Annu Rev Plant Biol.

[CR39] Cho K, Beom H-R, Jang Y-R, Altenbach SB, Vensel WH, Simon-Buss A (2018). Proteomic profiling and epitope analysis of the complex α-, γ- and ω-gliadin families in a commercial bread wheat. Front Plant Sci.

[CR40] Clarke BC, Maukai Y, Appels R (1996). The *Sec*-*1* locus on the short arm of chromosome 1R of rye (*Secale cereale*). Chromosoma.

[CR41] Colgrave ML, Goswami H, Byrne K, Blundell M, Howitt CA, Tanner GJ (2015). Proteomic profiling of 16 cereal grains and the application of targeted proteomics to detect wheat contamination. J Proteome Res.

[CR42] Delcour JA, Joye IJ, Pareyt B, Wilderjans E, Brijs K, Lagrain B (2012). Wheat gluten functionality as a quality determinant in cereal-based food products. Annu Rev Food Sci Technol.

[CR43] Dhaliwal AS, Mares DJ, Marshall DR (1990). Measurement of dough surface stickiness associated with the 1B/1R chromosome translocation in bread wheats. J Cereal Sci.

[CR44] Don C, Lichtendonk W, Plijter JJ, Hamer RJ (2003). Glutenin macropolymer: a gel formed by glutenin particles. J Cereal Sci.

[CR45] Don C, Lichtendonk WJ, Plijter JJ, Hamer RJ (2003). Understanding the link between GMP and dough: from glutenin particles in flour towards developed dough. J Cereal Sci.

[CR46] Don C, Mann G, Bekes F, Hamer RJ (2006). HMW-GS affect the properties of glutenin particles in GMP and thus flour quality. J Cereal Sci.

[CR47] Dong G, Ni Z, Yao Y, Nie X, Sun Q (2007). Wheat Dof transcription factor WPBF interacts with *TaQM* and activates transcription of an alpha-gliadin gene during wheat seed development. Plant Mol Biol.

[CR48] Dong LL, Zhang XF, Liu DC, Fan HJ, Sun JZ, Zhang ZJ (2010). New insights into the organization, recombination, expression and functional mechanism of low molecular weight glutenin subunit genes in bread wheat. PLoS ONE.

[CR49] Dong ZY, Yang YS, Li YW, Zhang KP, Lou HJ, An XL (2013). Haplotype variation of *Glu*-*D1* locus and the origin of *Glu*-*D1d* allele conferring superior end-use qualities in common wheat. PLoS ONE.

[CR50] Dong LL, Liu H, Zhang J, Yang S, Kong G, Chu JS (2015). Single-molecule real-time transcript sequencing facilitates common wheat genome annotation and grain transcriptome research. BMC Genomics.

[CR51] Dong LL, Huo N, Wang Y, Deal K, Wang D, Hu T (2016). Rapid evolutionary dynamics in a 2.8-Mb chromosomal region containing multiple prolamin and resistance gene families in *Aegilops tauschii*. Plant J.

[CR52] Dong ZY, Yang Y, Zhang K, Li Y, Wang J, Wang Z (2017). Development of a new set of molecular markers for examining *Glu*-*A1* variants in common wheat and ancestral species. PLoS ONE.

[CR53] D’Ovidio R, Masci S (2004). The low-molecular-weight glutenin subunits of wheat gluten. J Cereal Sci.

[CR54] Dupont FM, Vensel W, Encarnacao T, Chan R, Kasarda DD (2004). Similarities of omega gliadins from *Triticum urartu* to those encoded on chromosome 1A of hexaploid wheat and evidence for their post-translational processing. Theor Appl Genet.

[CR55] Dupont FM, Vensel WH, Tanaka CK, Hurkman WJ, Altenbach SB (2011). Deciphering the complexities of the wheat flour proteome using quantitative two-dimensional electrophoresis, three proteases and tandem mass spectrometry. Proteome Sci.

[CR56] Egidi E, Sestili F, Janni M, D’Ovidio R, Lafiandra D, Ceriotti A (2014). An asparagine residue at the N-terminus affects the maturation process of low molecular weight glutenin subunits of wheat endosperm. BMC Plant Biol.

[CR57] FAO (2017). The future of food and agriculture. Trends and challenges.

[CR58] Ferrante P, Masci S, D’Ovidio R, Lafiandra D, Volpi C, Mattei B (2006). A proteomic approach to verify in vivo expression of a novel gamma-gliadin containing an extra cysteine residue. Proteomics.

[CR59] Ferranti P, Mamone G, Picariello G, Addeo F (2007). Mass spectrometry analysis of gliadins in celiac disease. J Mass Spectrom.

[CR60] Fiedler KL, McGrath SC, Callahan JH, Ross MM (2014). Characterization of grain-specific peptide markers for the detection of gluten by mass spectrometry. J Agric Food Chem.

[CR61] Gaj R, Górski D, Przybył J (2013). Effect of differentiated phosphorus and potassium fertilization on winter wheat yield and quality. J Elementol.

[CR62] García-Molina MD, Giménez MJ, Sánchez-León S, Barro F (2019). Gluten free wheat: Are we there?. Nutrients.

[CR63] Geng Y, Pang B, Hao C, Tang S, Zhang X, Li T (2014). Expression of wheat high molecular weight glutenin subunit 1Bx is affected by large insertions and deletions located in the upstream flanking sequences. PLoS ONE.

[CR64] Gil-Humanes J, Pistón F, Hernando A, Álvarez JB, Shewry PR, Barro F (2008). Silencing of γ-gliadins by RNA interference (RNAi) in bread wheat. J Cereal Sci.

[CR65] Gil-Humanes J, Pistón F, Tollefsen S, Sollid LM, Francisco B (2010). Effective shutdown in the expression of celiac disease-related wheat gliadin T-cell epitopes by RNA interference. Proc Natl Acad Sci USA.

[CR66] Gil-Humanes J, Pistón F, Giménez MJ, Martín A, Barro F (2012). The introgression of RNAi silencing of γ-gliadins into commercial lines of bread wheat changes the mixing and technological properties of the dough. PLoS ONE.

[CR67] Gil-Humanes J, Pistón F, Barro F, Rosell CM (2014). The shutdown of celiac disease-related gliadin epitopes in bread wheat by RNAi provides flours with increased stability and better tolerance to over-mixing. PLoS ONE.

[CR68] Gil-Humanes J, Pistón F, Altamirano-Fortoul R, Real A, Comino I, Sousa C (2014). Reduced-gliadin wheat bread: an alternative to the gluten-free diet for consumers suffering gluten-related pathologies. PLoS ONE.

[CR69] Gilissen LJWJ, Van der Meer IM, Smulders MJM (2014). Reducing the incidence of allergy and intolerance to cereals. J Cereal Sci.

[CR70] Gobaa S, Bancel E, Kleijer G, Stamp P, Branlard G (2007). Effect of the 1BL.1RS translocation on the wheat endosperm, as revealed by proteomic analysis. Proteomics.

[CR71] Gras PW, Anderssen RS, Keentok M, Békés F, Appels R (2001). Gluten protein functionality in wheat flour processing: a review. Aust J Agric Res.

[CR72] Graybosch RA (2001). Uneasy unions: quality effects of rye chromatin transfers to wheat. J Cereal Sci.

[CR73] Grove H, Hollung K, Moldestad A, Færgestad EM, Uhlen AK (2009). Proteome changes in wheat subjected to different nitrogen and sulfur fertilizations. J Agric Food Chem.

[CR74] Gu YQ, Salse J, Coleman-Derr D, Dupin A, Crossman C, Lazo GR (2006). Types and rates of sequence evolution at the high-molecular-weight glutenin locus in hexaploid wheat and its ancestral genomes. Genetics.

[CR75] Guo W, Yang H, Liu Y, Gao Y, Ni Z, Peng H (2015). The wheat transcription factor TaGAMyb recruits histone acetyltransferase and activates the expression of a high-molecular-weight glutenin subunit gene. Plant J.

[CR76] Gupta RB, Khan K, MacRitchie F (1993). Biochemical basis of flour properties in bread wheats. I. Effects of variation in quantity and size distribution of polymeric proteins. J Cereal Sci.

[CR77] Huo N, Dong L, Zhang S, Wang Y, Zhu T, Mohr T (2017). New insights into structural organization and gene duplication in a 1.75-Mb genomic region harboring the α-gliadin gene family in *Aegilops tauschii*, the source of wheat D genome. Plant J.

[CR78] Huo N, Zhang S, Zhu T, Dong L, Mohr T, Hu T (2018). Gene duplication and evolution dynamics in the homoeologous regions harboring multiple prolamin and resistance gene families in hexaploid wheat. Front Plant Sci.

[CR79] Huo N, Zhu T, Altenbach S, Dong L, Wang Y, Mohr T (2018). Dynamic evolution of α-gliadin prolamin gene family in homoeologous genomes of hexaploid wheat. Sci Rep.

[CR80] Hurkman WJ, Tanaka CK, Vensel WH, Thilmony R, Altenbach SB (2013). Comparative proteomic analysis of the effect of temperature and fertilizer on gliadin and glutenin accumulation in the developing endosperm and flour from *Triticum aestivum* L. cv. Butte 86. Proteome Sci.

[CR81] Johansson E, Malik AH, Hussain A, Rasheed F, Newson WR, Plivelic T (2013). Wheat gluten polymer structures: the impact of genotype, environment, and processing on their functionality in various applications. Cereal Chem.

[CR82] Jouanin A, Schaart JG, Boyd LA, Cockram J, Leigh FJ, Bates R (2019). Outlook for coeliac disease patients: towards bread wheat with hypoimmunogenic gluten by gene editing of α- and γ-gliadin gene families. BMC Plant Biol.

[CR83] Jouanin A, Tenorio-Berrio R, Schaart JG, Leigh F, Visser RGF, Smulders JM (2019). Optimization of droplet digital PCR for determining copy number variation of α-gliadin genes in mutant and gene-edited polyploid bread wheat. J Cereal Sci.

[CR84] Juhász A, Makai S, Sebestyén E, Tamás L, Balázs E (2011). Role of conserved non-coding regulatory elements in LMW glutenin gene expression. PLoS ONE.

[CR85] Juhász A, Belova T, Florides CG, Maulis C, Fischer I, Gell G (2018). Genome mapping of seed-borne allergens and immunoresponsive proteins in wheat. Sci Adv.

[CR86] Kan YC, Wan YF, Beaudoin F, Leader DJ, Edwards K, Poole R (2006). Transcriptome analysis reveals differentially expressed storage protein transcripts in seeds of *Aegilops* and wheat. J Cereal Sci.

[CR87] Kasarda DD, Adalsteins E, Lew EJ, Lazo GR, Altenbach SB (2013). Farinin: characterization of a novel wheat endosperm protein belonging to the prolamin superfamily. J Agric Food Chem.

[CR88] Kawaura K, Mochida K, Ogihara Y (2005). Expression profile of two storage-protein gene families in hexaploid wheat revealed by large-scale analysis of expressed sequence tags. Plant Physiol.

[CR89] Knott GJ, Doudna JA (2018). CRISPR-Cas guides the future of genetic engineering. Science.

[CR90] Li MJ, Li YQ, Zhang N, Shi ZL (2016). Cloning of the ω-secalin gene family in a wheat 1BL/1RS translocation line using BAC clone sequencing. Electron J Biotechnol.

[CR91] Li D, Jin H, Zhang K, Wang Z, Wang F, Zhao Y (2018). Analysis of the *Gli*-*D2* locus identifies a genetic target for simultaneously improving the breadmaking and health-related traits of common wheat. Plant J.

[CR92] Li J, Wang K, Li G, Li Y, Zhang Y, Liu Z (2019). Dissecting conserved *cis*-regulatory modules of *Glu*-*1* promoters which confer the highly active endosperm-specific expression via stable wheat transformation. Crop J.

[CR93] Ling HQ, Ma B, Shi XL, Liu H, Dong LL, Sun H (2018). Genome sequence of the progenitor of wheat A subgenome *Triticum urartu*. Nature.

[CR94] Lionetti E, Gatti S, Pulvirenti A, Catassi C (2015). Celiac disease from a global perspective. Best Pract Res Clin Gastroenterol.

[CR95] Liu L, Ikeda TM, Branlard G, Peña RJ, Rogers WJ, Lerner SE (2010). Comparison of low molecular weight glutenin subunits identified by SDS-PAGE, 2-DE, MALDI-TOF-MS and PCR in common wheat. BMC Plant Biol.

[CR96] Luo MC, Gu YQ, Puiu D, Wang H, Twardziok SO, Deal KR (2017). Genome sequence of the progenitor of the wheat D genome *Aegilops tauschii*. Nature.

[CR97] Ma F, Li M, Li T, Liu W, Liu Y, Li Y (2013). Overexpression of avenin-like b proteins in bread wheat (*Triticum aestivum* L.) improves dough mixing properties by their incorporation into glutenin polymers. PLoS One.

[CR98] Ma F, Li M, Yu L, Li Y, Liu Y, Li T (2013). Transformation of common wheat (*Triticum aestivum* L.) with avenin-like b gene improves flour mixing properties. Mol Breed.

[CR99] Ma C, Zhou J, Chen G, Bian Y, Lv D, Li X (2014). iTRAQ-based quantitative proteome and phosphoprotein characterization reveals the central metabolism changes involved in wheat grain development. BMC Genomics.

[CR100] Ma W, Yu Z, She M, Zhao Y, Islam S (2019). Wheat gluten protein and its impact on wheat processing quality. Front Agric Sci Eng.

[CR101] MacRitchie F (2014). Theories of glutenin/dough systems. J Cereal Sci.

[CR102] Mamone G, Addeo F, Chianese L, Di Luccia A, De Martino A, Nappo A (2005). Characterization of wheat gliadin proteins by combined two-dimensional gel electrophoresis and tandem mass spectrometry. Proteomics.

[CR103] Mamone G, De Caro S, Di Luccia A, Addeo F, Ferranti P (2009). Proteomic-based analytical approach for the characterization of glutenin subunits in durum wheat. J Mass Spectrom.

[CR104] Martínez-Esteso MJ, Nørgaard J, Brohée M, Haraszi R, Maquet A, O’Connor G (2016). Defining the wheat gluten peptide fingerprint via a discovery and targeted proteomics approach. J Proteom.

[CR105] Matsuo H, Morita E, Tatham AS, Morimoto K, Horikawa T, Osuna H (2004). Identification of the IgE-binding epitope in omega-5 gliadin, a major allergen in wheat-dependent exercise-induced anaphylaxis. J Biol Chem.

[CR106] Matsuo H, Kohno K, Niihara H, Morita E (2005). Specific IgE determination to epitope peptides of omega-5 gliadin and high molecular weight glutenin subunit is a useful tool for diagnosis of wheat-dependent exercise-induced anaphylaxis. J Immunol.

[CR107] Mishra R, Joshi RK, Zhao K (2019). Base editing in crops: current advances, limitations and future implications. Plant Biotechnol J.

[CR108] Moehs CP, Austill WJ, Holm A, Large TAG, Loeffler D, Mullenberg J (2019). Development of decreased-gluten wheat enabled by determination of the genetic basis of *lys3a* barley. Plant Physiol.

[CR109] Muccilli V, Cunsolo V, Saletti R, Foti S, Masci S, Lafinadra D (2005). Characterization of B- and C-type low molecular weight glutenin subunits by electrospray ionization mass spectrometry and matrix-assisted laser desorption/ionization mass spectrometry. Proteomics.

[CR110] Mueller E, Wieser H, Koehler P (2016). Preparation and chemical characterization of glutenin macropolymer (GMP) gel. J Cereal Sci.

[CR111] Naeem HA, MacRitchie F (2005). Polymerization of glutenin during grain development in near-isogenic wheat lines differing at *Glu*-*D1* and *Glu*-*B1* in greenhouse and field. J Cereal Sci.

[CR112] Ni Y, Yang D, Wang Z, Yin Y, Cai T, Dai Z (2014). Phosphorus affects high-molecular-weight glutenin subunits and glutenin macropolymer size distribution in wheat grains. J Agric Sci.

[CR113] Noma S, Kawaura K, Hayakawa K, Abe C, Tsuge N, Ogihara Y (2016). Comprehensive molecular characterization of the α/β-gliadin multigene family in hexaploid wheat. Mol Genet Genomics.

[CR114] Nunes-Miranda JD, Bancel E, Viala D, Chambon C, Capelo JL, Branlard G (2017). Wheat glutenin: the “tail” of the 1By protein subunits. J Proteome.

[CR115] Osorio CE, Wen N, Mejias JH, Liu B, Reinbothe S, von Wettstein D (2019). Development of wheat genotypes expressing a glutamine-specific endoprotease from barley and a prolyl endopeptidase from *Flavobacterium meningosepticum* or *Pyrococcus furiosus* as a potential remedy to celiac disease. Funct Integr Genomics.

[CR116] Pistón F, Gil-Humanes J, Rodríguez-Quijano M, Barro F (2011). Down-regulating γ-gliadins in bread wheat leads to non-specific increases in other gluten proteins and has no major effect on dough gluten strength. PLoS ONE.

[CR117] Plessis A, Ravel C, Bordes J, Balfourier F, Martre P (2013). Association study of wheat grain protein composition reveals that gliadin and glutenin composition are trans-regulated by different chromosome regions. J Exp Bot.

[CR118] Rasheed A, Xia XC, Yan YM, Appels R, Mahmood T, He ZH (2014). Wheat seed storage proteins: advances in molecular genetics, diversity and breeding applications. J Cereal Sci.

[CR119] Ravel C, Nagy IJ, Martre P, Sourdille P, Dardevet M, Balfourier F (2006). Single nucleotide polymorphism, genetic mapping, and expression of genes coding for the DOF wheat prolamin-box binding factor. Funct Integr Genomics.

[CR120] Ravel C, Martre P, Romeuf I, Dardevet M, El-Malki R, Bordes J (2009). Nucleotide polymorphism in the wheat transcriptional activator Spa influences its pattern of expression and has pleiotropic effects on grain protein composition, dough viscoelasticity, and grain hardness. Plant Physiol.

[CR121] Ravel C, Fiquet S, Boudet J, Dardevet M, Vincent J, Merlino M (2014). Conserved *cis*-regulatory modules in promoters of genes encoding wheat high-molecular-weight glutenin subunits. Front Plant Sci.

[CR122] Ribeiro M, Nunes-Miranda JD, Branlard G, Carrillo JM, Rodriguez-Quijano M, Igrejas G (2013). One hundred years of grain omics: identifying the glutens that feed the world. J Proteome Res.

[CR123] Roy N, Islam S, Yu Z, Lu M, Lafiandra D, Zhao Y (2019). Introgression of an expressed HMW 1Ay glutenin subunit allele into bread wheat cv. Lincoln increases grain protein content and breadmaking quality without yield penalty. Theor Appl Genet.

[CR124] Rustgi S, Shewry P, Brouns F, Deleu L, Delcour JA (2019). Wheat seed proteins: factors influencing their content, composition, and technological properties, and strategies to reduce adverse reactions. Compr Rev Food Sci F.

[CR125] Salentijn EM, Goryunova SV, Bas N, van der Meer IM, van den Broeck HC, Bastien T (2009). Tetraploid and hexaploid wheat varieties reveal large differences in expression of alpha-gliadins from homoeologous *Gli*-*2* loci. BMC Genom.

[CR126] Sánchez-León S, Gil-Humanes J, Ozuna CV, Giménez MJ, Sousa C, Voytas DF (2018). Low-gluten, nontransgenic wheat engineered with CRISPR/Cas9. Plant Biotechnol J.

[CR127] Schalk K, Lexhaller B, Koehler P, Scherf KA (2017). Isolation and characterization of gluten protein types from wheat, rye, barley and oats for use as reference materials. PLoS ONE.

[CR128] Scherf KA, Koehler P, Wieser H (2016). Gluten and wheat sensitivities—an overview. J Cereal Sci.

[CR129] Shan L, Molberg O, Parrot I, Hausch F, Filia F, Gray GM (2002). Structural basis for gluten intolerance in celiac sprue. Science.

[CR130] She M, Ye X, Yan Y, Howit C, Belgard M, Ma W (2011). Gene networks in the synthesis and deposition of protein polymers during grain development of wheat. Funct Integr Genomics.

[CR131] Shewry PR (2019). What is gluten—Why is it special?. Front Nutr.

[CR132] Shewry PR, Hey SJ (2015). The contribution of wheat to human diet and health. Food Energy Secur.

[CR133] Shewry PR, Tatham AS (2016). Improving wheat to remove coeliac epitopes but retain functionality. J Cereal Sci.

[CR134] Shewry PR, Halford NG, Lafiandra D (2003). Genetics of wheat gluten proteins. Adv Genet.

[CR135] Shiferaw B, Smale M, Braun HJ, Duveiller E, Reynolds M, Muricho G (2013). Crops that feed the world 10. Past successes and future challenges to the role played by wheat in global food security. Food Secur.

[CR136] Sun FS, Liu X, Wei QH, Liu JN, Yang TX, Jia LY (2017). Functional characterization of TaFUSCA3, a B3-superfamily transcription factor gene in the wheat. Front Plant Sci.

[CR137] The International Wheat Genome Sequencing Consortium (IWGSC) (2018). Shifting the limits in wheat research and breeding using a fully annotated reference genome. Science.

[CR138] Thomas MS, Flavell RB (1990). Identification of an enhancer element for the endosperm-specific expression of high molecular weight glutenin. Plant Cell.

[CR139] Tronsmo KM, Færgestad EM, Longva A, Schofield JD, Magnus EM (2002). A study of how size distribution of gluten proteins, surface properties of gluten and dough mixing properties relate to baking properties of wheat flours. J Cereal Sci.

[CR140] Tye-Din JA, Stewart JA, Dromey JA, Beissbarth T, van Heel DA, Tatham A (2010). Comprehensive, quantitative mapping of T cell epitopes in gluten in celiac disease. Sci Transl Med.

[CR141] Uauy C (2017). Wheat genomics comes of age. Curr Opin Plant Biol.

[CR142] Uvackova L, Skultety L, Bekesova S, McClain S, Hajduch M (2013). MS(E) based multiplex protein analysis quantified important allergenic proteins and detected relevant peptides carrying known epitopes in wheat grain extracts. J Proteome Res.

[CR143] van den Broeck HC, van Herpen TW, Schuit C, Salentijn EM, Dekking L, Bosch D (2009). Removing celiac disease-related gluten proteins from bread wheat while retaining technological properties: a study with Chinese Spring deletion lines. BMC Plant Biol.

[CR144] van Herpen TWJM, Goryunova SV, Van der Schoot J, Mitreva M, Salentijn EMJ, Vorst O (2006). Alpha-gliadin genes from the A, B and D genomes of wheat contain different sets of celiac disease epitopes. BMC Genom.

[CR145] van Herpen TWJM, Cordewener JHG, Klok HJ, Freeman J, America AHP, Bosch D (2008). The origin and early development of wheat glutenin particles. J Cereal Sci.

[CR146] van Herpen TWJM, Riley M, Sparks C, Jones HD, Gritsch C, Dekking EH (2008). Detailed analysis of the expression of an alpha-gliadin promoter and the deposition of alpha-gliadin protein during wheat grain development. Ann Bot.

[CR147] Vensel WH, Tanaka CK, Altenbach SB (2014). Protein composition of wheat gluten polymer fractions determined by quantitative two-dimensional gel electrophoresis and tandem mass spectrometry. Proteome Sci.

[CR148] Waga J, Skoczowski A (2014). Development and characteristics of ω-gliadin-free wheat genotypes. Euphytica.

[CR149] Waga J, Zientarski J, Szaleniec M, Obtulowicz K, Dyga W, Skoczowski A (2013). Null alleles in gliadin coding loci and wheat allergenic properties. Am J Plant Sci.

[CR150] Wan YF, Poole RL, Huttly AK, Toscano-Underwood C, Feeney K, Welham S (2008). Transcriptome analysis of grain development in hexaploid wheat. BMC Genom.

[CR151] Wan YF, Shewry PR, Hawkesford MJ (2013). A novel family of γ-gliadin genes are highly regulated by nitrogen supply in developing wheat grain. J Exp Bot.

[CR152] Wang K, Zhang X, Zhao Y, Chen F, Xia G (2013). Structure, variation and expression analysis of glutenin gene promoters from *Triticum aestivum* cultivar Chinese Spring shows the distal region of promoter 1Bx7 is key regulatory sequence. Gene.

[CR153] Wang DW, Li D, Wang J, Zhao Y, Wang Z, Yue G (2017). Genome-wide analysis of complex wheat gliadins, the dominant carriers of celiac disease epitopes. Sci Rep.

[CR154] Wen S, Wen N, Pang J, Langen G, Brew-Appiah RA, Mejias JH (2012). Structural genes of wheat and barley 5-methylcytosine DNA glycosylases and their potential applications for human health. Proc Natl Acad Sci USA.

[CR155] Wieser H (2007). Chemistry of gluten proteins. Food Microbiol.

[CR156] Wrigley CW, Asenstorfer R, Batey IL, Cornish GB, Day L, Mares D, Carver BF (2009). The biochemical and molecular basis of wheat quality. Wheat science and trade.

[CR157] Xue C, Schulte auf’m Erley G, Rossmann A, Schuster R, Koehler P, Mühling KH (2016). Split nitrogen application improves wheat baking quality by influencing protein composition rather than concentration. Front Plant Sci.

[CR158] Xue C, Matros A, Mock HP, Mühling KH (2019). Protein composition and baking quality of wheat flour as affected by split nitrogen application. Front Plant Sci.

[CR159] Yamamoto M, Mukai Y (2005). High-resolution physical mapping of the *secalin*-*1* locus of rye on extended DNA fibers. Cytogenet Genome Res.

[CR160] Yang F, Jørgensen AD, Li H, Søndergaard I, Finnie C, Svensson B (2011). Implications of high-temperature events and water deficits on protein profiles in wheat (*Triticum aestivum* L. cv. Vinjett) grain. Proteomics.

[CR161] Yin K, Gao C, Qiu JL (2017). Progress and prospects in plant genome editing. Nat Plants.

[CR162] Yu X, Chen X, Wang L, Yang Y, Zhu X, Shao S (2017). Novel insights into the effect of nitrogen on storage protein biosynthesis and protein body development in wheat caryopsis. J Exp Bot.

[CR163] Yu Z, Islam S, She M, Diepeveen D, Zhang Y, Tang G (2018). Wheat grain protein accumulation and polymerization mechanisms driven by nitrogen fertilization. Plant J.

[CR164] Zevallos VF, Raker V, Tenzer S, Jimenez-Calvente C, Ashfaq-Khan M, Rüssel N (2017). Nutritional wheat amylase-trypsin inhibitors promote intestinal inflammation via activation of myeloid cells. Gastroenterology.

[CR165] Zhang M, Ma CY, Lv DW, Zhen SM, Li XH, Yan YM (2014). Comparative phosphoproteome analysis of the developing grains in bread wheat (*Triticum aestivum* L.) under well-watered and water-deficit conditions. J Proteome Res.

[CR166] Zhang X, Shi Z, Tian Y, Zhou Q, Cai J, Dai T (2016). Salt stress increases content and size of glutenin macropolymers in wheat grain. Food Chem.

[CR167] Zhang Y, Hu X, Islam S, She M, Peng Y, Yu Z (2018). New insights into the evolution of wheat avenin-like proteins in wild emmer wheat (*Triticum dicoccoides*). Proc Natl Acad Sci USA.

[CR168] Zhang YF, Lou H, Guo D, Zhang R, Su M, Hou Z (2018). Identifying changes in the wheat kernel proteome under heat stress using iTRAQ. Crop J.

[CR169] Zhao Q, Li Y, Li WY, Wang P, Chen XG, Yin YP (2011). Effects of water-nitrogen interaction on content of high molecular weight glutenin subunits and GMP size distribution in wheat cultivars of different genotypes. Sci Agric Sin.

[CR170] Zhao G, Zou C, Li K, Wang K, Li T, Gao L (2017). The *Aegilops tauschii* genome reveals multiple impacts of transposons. Nat Plants.

[CR171] Zhen S, Deng X, Zhang M, Zhu G, Lv D, Wang Y (2017). Comparative phosphoproteomic analysis under high-nitrogen fertilizer reveals central phosphoproteins promoting wheat grain starch and protein synthesis. Front Plant Sci.

[CR172] Zhen S, Deng X, Li M, Zhu D, Yan Y (2018). 2D-DIGE comparative proteomic analysis of developing wheat grains under high-nitrogen fertilization revealed key differentially accumulated proteins that promote storage protein and starch biosyntheses. Anal Bioanal Chem.

[CR173] Zheng T, Qi PF, Cao YL, Han YN, Ma HL, Guo ZR (2018). Mechanisms of wheat (*Triticum aestivum*) grain storage proteins in response to nitrogen application and its impacts on processing quality. Sci Rep.

[CR174] Zhong Y, Wang W, Huang X, Liu M, Hebelstrup KH, Yang D (2019). Nitrogen topdressing timing modifies the gluten quality and grain hardness related protein levels as revealed by iTRAQ. Food Chem.

[CR175] Zhou J, Liu D, Deng X, Zhen S, Wang Z, Yan Y (2018). Effects of water deficit on breadmaking quality and storage protein compositions in bread wheat (*Triticum aestivum* L.). J Sci Food Agric.

[CR176] Zhu J, Fang L, Yu J, Zhao Y, Chen F, Xia G (2018). 5-Azacytidine treatment and *TaPBF*-*D* over-expression increases glutenin accumulation within the wheat grain by hypomethylating the *Glu*-*1* promoters. Theor Appl Genet.

[CR177] Zörb C, Grover C, Steinfurth D, Mühling KH (2010). Quantitative proteome analysis of wheat gluten as influenced by N and S nutrition. Plant Soil.

[CR178] Zörb C, Becker D, Hasler M, Mühling KH, Gödde V, Niehaus K (2013). Silencing of the sulfur rich α-gliadin storage protein family in wheat grains (*Triticum aestivum* L.) causes no unintended side-effects on other metabolites. Front Plant Sci.

[CR179] Zörb C, Ludewig U, Hawkesford MJ (2018). Perspective on wheat yield and quality with reduced nitrogen supply. Trends Plant Sci.

